# Supplementary material for: Large-scale cryovolcanic resurfacing on Pluto
Source: Nat Commun. 2022 Mar 29;13:1542. doi: 10.1038/s41467-022-29056-3 (PMC8964750; doi:10.1038/s41467-022-29056-3)
Supplement: Supplementary file 1 — Supplementary information [file 41467_2022_29056_MOESM1_ESM.pdf]

Supplement: Large-scale cryovolcanic resurfacing on Pluto

Singer et al., Nature Communications

Supplementary Note 1: New Horizons Image Data

Wright and Piccard were viewed in several datasets listed in Supplementary Table 1. The best combination of lighting, resolution, and signal-to-noise comes from the ~315 m px^{-1} Multi-spectral Visible Imaging Camera (MVIC) scan taken on closest approach. We used all available imaging of the Wright and Piccard region with different lighting geometries and also topographic products[1] when examining the morphology of this region. We also used simulated hillshades (shaded relief maps) to check for biases introduced by specific lighting conditions.

Supplementary Table 1: High-resolution New Horizons image data for Pluto

Request ID*	Instrument†	Instrument Mode	Pixel Scale [m px^{-1}]	Mosaic size or Scan‡	Exposure or Scan rate
PELR_P_LORRI	LORRI	1×1	850 ± 30	4×5	150 ms
PELR_P_LEISA_HIRES‡	LORRI	1×1	234 ± 13	1×12	50 ms
PELR_P_MPAN_1‡	LORRI	1×1	117 ± 2	1×27	10 ms
PELR_P_ MVIC_ LORRI_CA‡	LORRI	1×1	76	1x35	10 ms
PEMV_P_MPAN1	MVIC	Pan TDI 1	480 ± 5	Scan	1600 µrad s^{-1}
PEMV_P_ MVIC_ LORRI_CA	MVIC	Pan TDI 2	315 ± 8	Scan	1000 µrad s^{-1}
PEMV_P_Color2	MVIC	Color	660	Scan	1045.5 µrad s^{-1}
PELE_01_P_LEISA_Alice_2a	LEISA		~7,100	Scan	105.1 µrad s^{-1}
PELE_01_P_LEISA_Alice_2b	LEISA		~6,400	Scan	105.1 µrad s^{-1}
PELE_01_P_LEISA_Hires	LEISA		~3,100	Scan	105.1 µrad s^{-1}

*The Request ID are unique identifiers for each observation stored at NASA's Planetary Data System (https://pds-smallbodies.astro.umd.edu/data_sb/missions/newhorizons/index.shtml).
†For more information about the New Horizons instruments, please see [2, 3]
‡Given in number of instrument field-of-view footprints across the mosaic.
Note: All observations listed here were taken within a short time of each other near the closest approach of the New Horizons spacecraft when the sub solar point was at ~128.3°E, 51.6°N.

Supplementary Note 2: Regional Context of the Wright and Piccard Montes Region

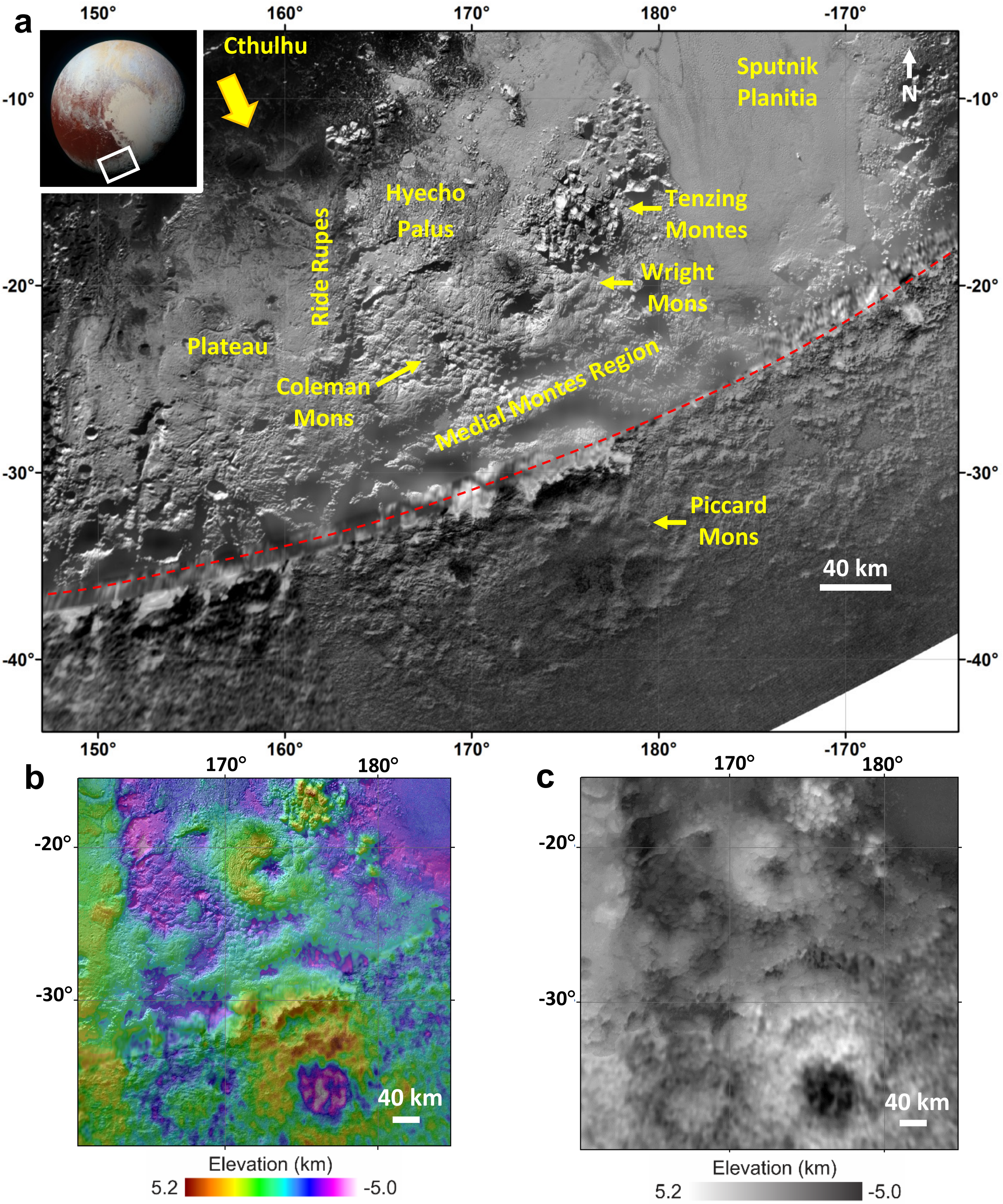

Supplementary Figure 1 | Overview of putative cryovolcanic terrains. **a,** Panchromatic basemap mosaic (image resolutions ranging from ~235-480 m px^{-1}; see Supplementary Table 1); dashed red curve indicates the transition from directly sunlit terrain to haze-lit terrain, **b,** color

topography overlain on basemap [1], and **c,** greyscale topography alone. Simple cylindrical projection. All images in the supplement are shown with north up, and the lighting direction is the same as indicated here with the large arrow at the upper left unless otherwise indicated.

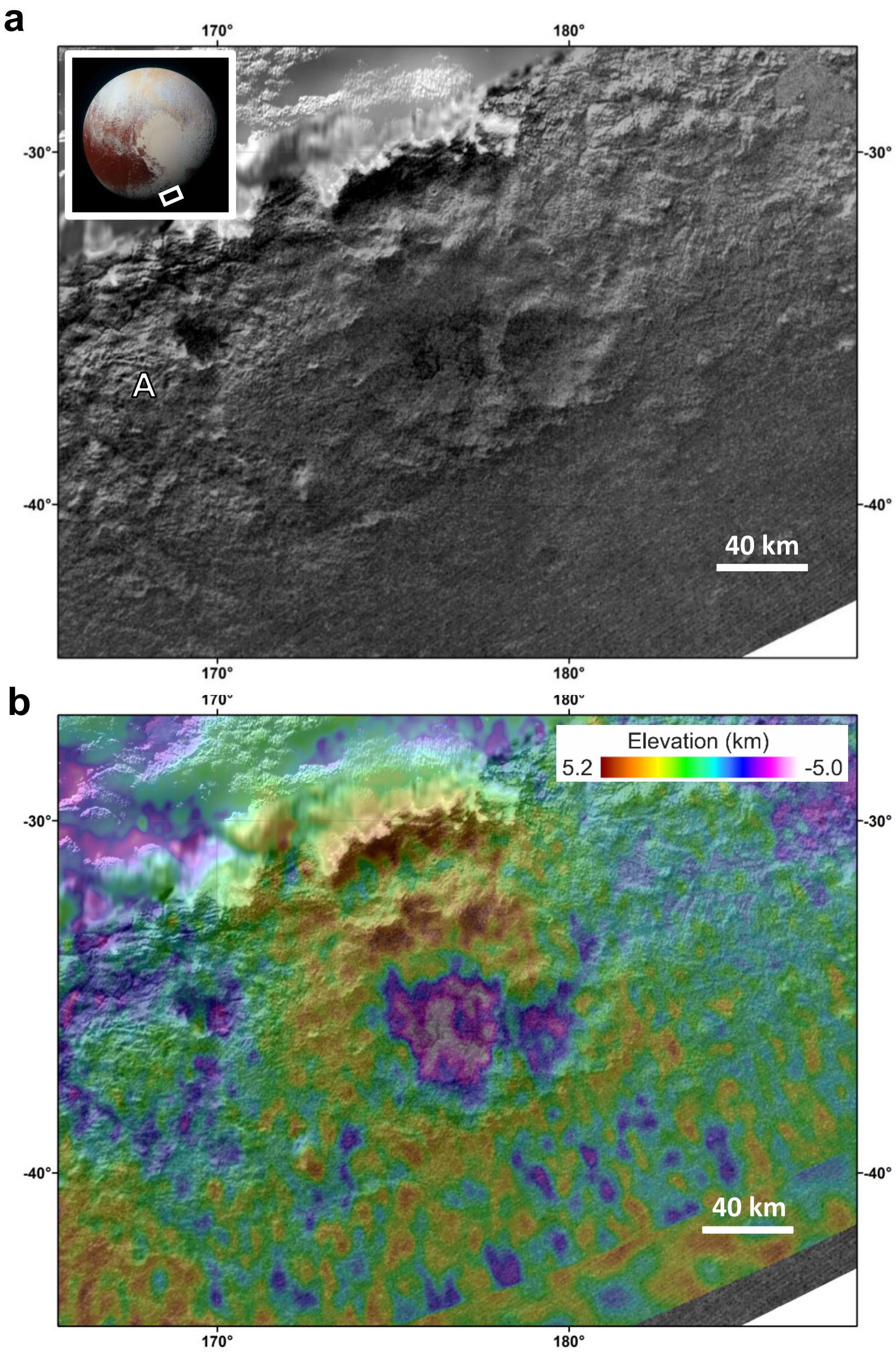

Supplementary Figure 2 | Piccard Mons and surrounding terrain. (a) Panchromatic base image (b) topography overlain on basemap. This image is lit by haze light reflected out of Pluto's thin atmosphere.

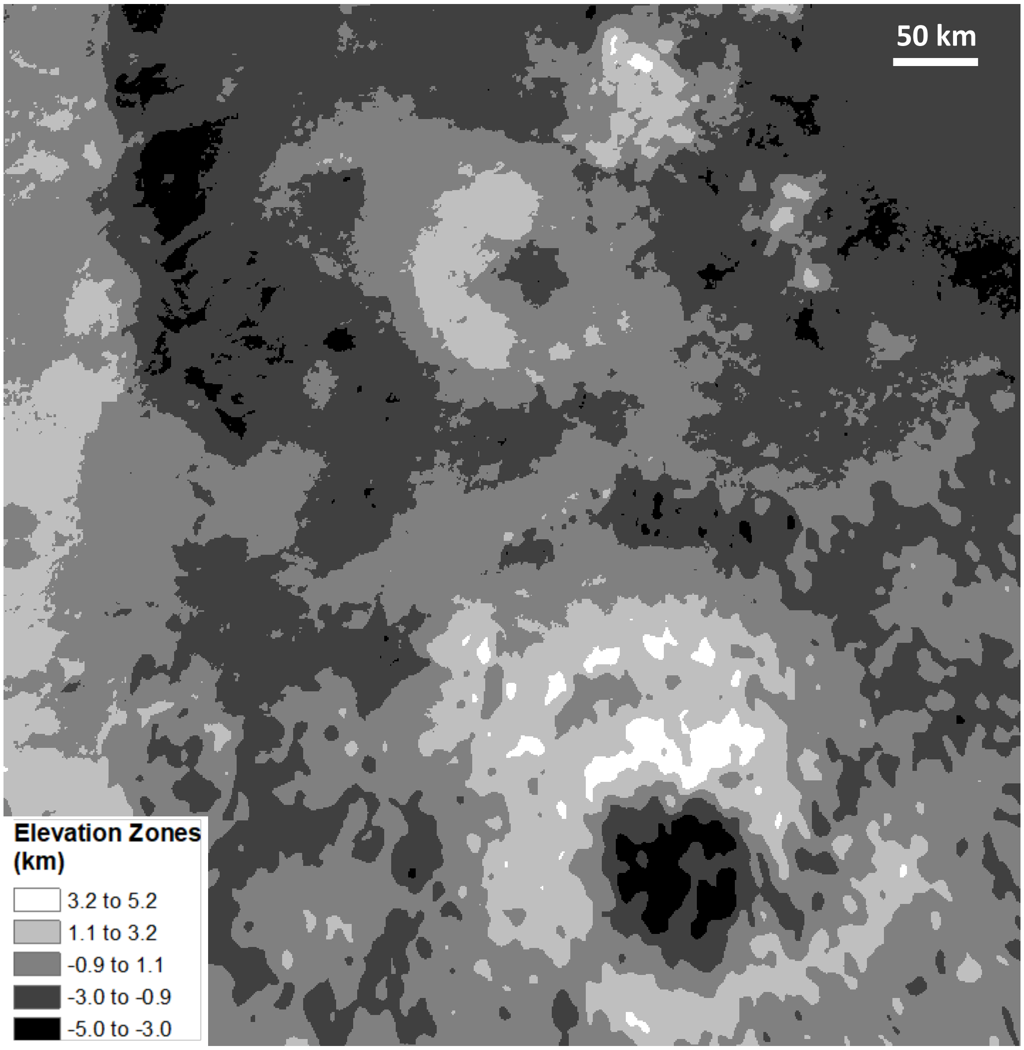

Supplementary Figure 3 | The topography of the Wright and Piccard regions here is split into five equal-sized height contours where white is the highest and black is the lowest. This view shows the gross structure of the region and how the "summit" regions around Wright and Piccard are not continuous, and also how the large rise in the medial montes region is connected to both Wright and Piccard Montes. The longitude and latitude extents of the figure are ~160–175°E and ~15–40°S.

Supplementary Note 3: Hummocky/undulating terrain wavelength and topography

Here we present some additional information about the undulating/hummocky terrain on the flanks of Wright Mons and the surrounding terrain. The topographic profiles show the variety of morphological expressions of the lumpy terrain (Supplementary Figure 4). Some hummocks have a narrower-sharper boundary between themselves and adjacent hummocks, while others have very shallow or gradual transitions with their neighboring features. However, it is still clear that the hummocks are often not simple individual hills, but are more interconnected to their surroundings and many have one or more "sides" that have no distinct boundary. It is possible that some of the sharper transitions represent the toes of lobate flow fronts, but the oblique lighting (<30° elevation angle) strongly affects the appearance of features in this region.

Thus, we have performed mapping on both the panchromatic image base and the highest resolution topographic product that covers the region. The topography used below is a stereo product produced from an ~315 m px^{-1} MVIC scan and an ~240 m px^{-1} LORRI ride-along mosaic with a vertical precision of 90 m[1] (also see methods). The base image used below for "visual mapping" is the ~315 m px^{-1} MVIC scan which has better signal-to-noise and less smear than the higher resolution 240 m px^{-1} LORRI mosaic.

All mapping involves some subjectivity, but here we attempted to characterize the general wavelength or size of the hummocky-like terrain using a simple metric that can be applied fairly consistently to many of the features. Here we measured across the convex surface of the bulbous mounds or flows in cases where at least two sides of the feature appeared to be bounded by troughs (Supplementary Figure 5). Because the lumpy terrain often does not represent individual hills/hummocks (as described above), we do not attempt to map their full circumference at their base, as this would be quite arbitrary and impossible to consistently define in many cases. We generally avoided the heavily shadowed regions, although the boundaries of the hummocks can be revealed to a degree by stretching the image (here we used built-in ArcGIS routines that optimize the image viewing for low-light regions, rather than bright regions). Although the image in Supplementary Figure 5 is stretched to reveal the brighter portions of the scene, some of the lines extend into the shadows because we were able to see the transitions between features there in a different stretch of the image. The feature edges are more well-defined where they are perpendicular to the direction of the incoming light (i.e., feature edges that trend ~NE-SW are more easily seen), thus more features were measured that trend this direction in the panchromatic image.

Both measurement sets, the panchromatic mapping and the topographic mapping, produced similar results in that most features measured have a wavelength of ~6 and 14 km, with a broad peak between 6-12 km. The western side of this region generally has larger hummocks. The topography shows the hummocks on the northern flank of Wright Mons that are hidden by the oblique lighting in the panchromatic view.

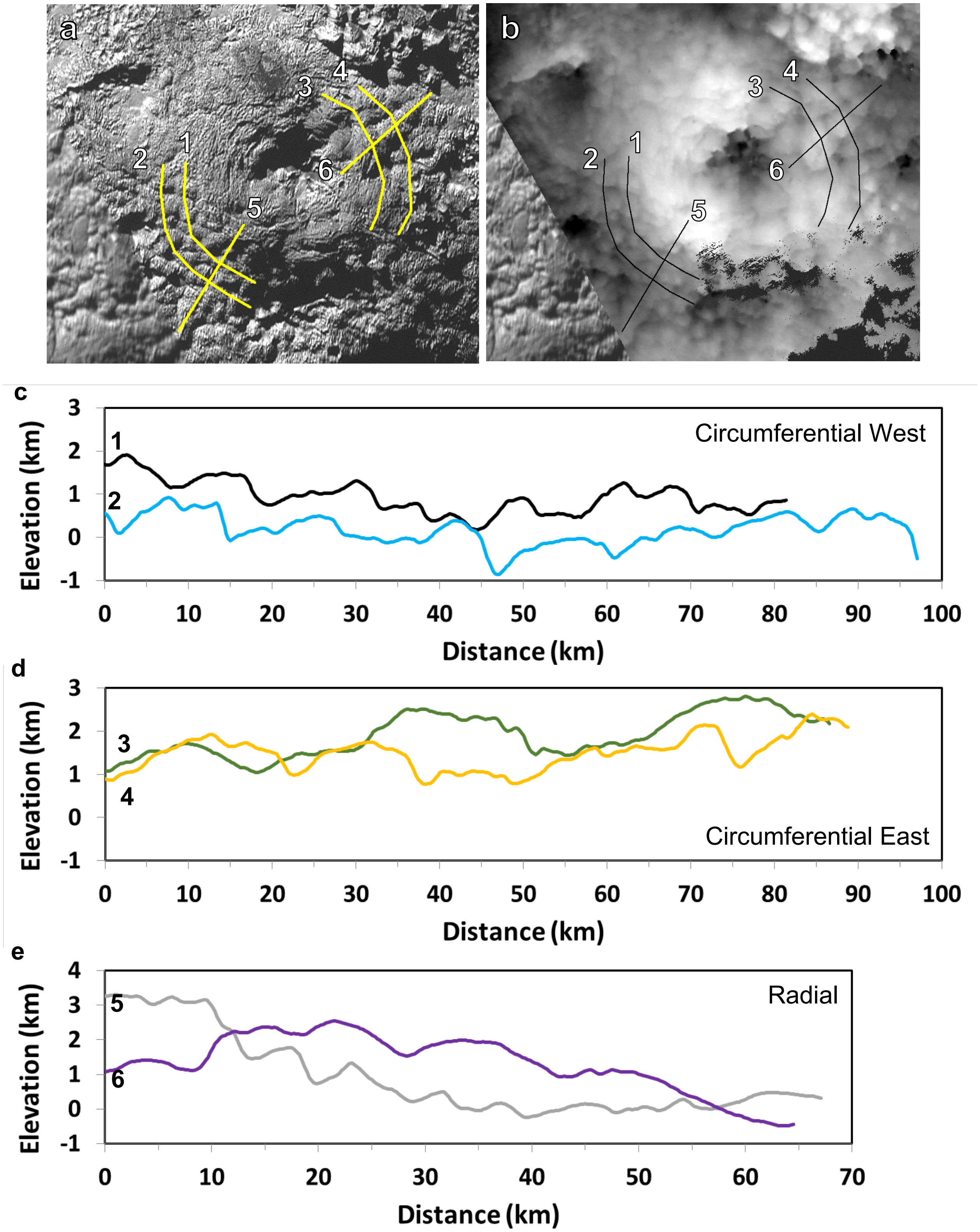

Supplementary Figure 4 | Topographic profiles of hummocks. (a) panchromatic image and (b) topography for Wright Mons with topographic profile lines over the undulating/hummocky terrain.

(c-e) Topographic profiles as shown in panels a-b illustrate the varied sizes and shapes of the lumps and depressions between them. Profiles start at the numbered end of each line. The mounds on the eastern side of Wright Mons are larger on average. The longitude and latitude extents of both panels are ~165–177°E and ~17–26°S.

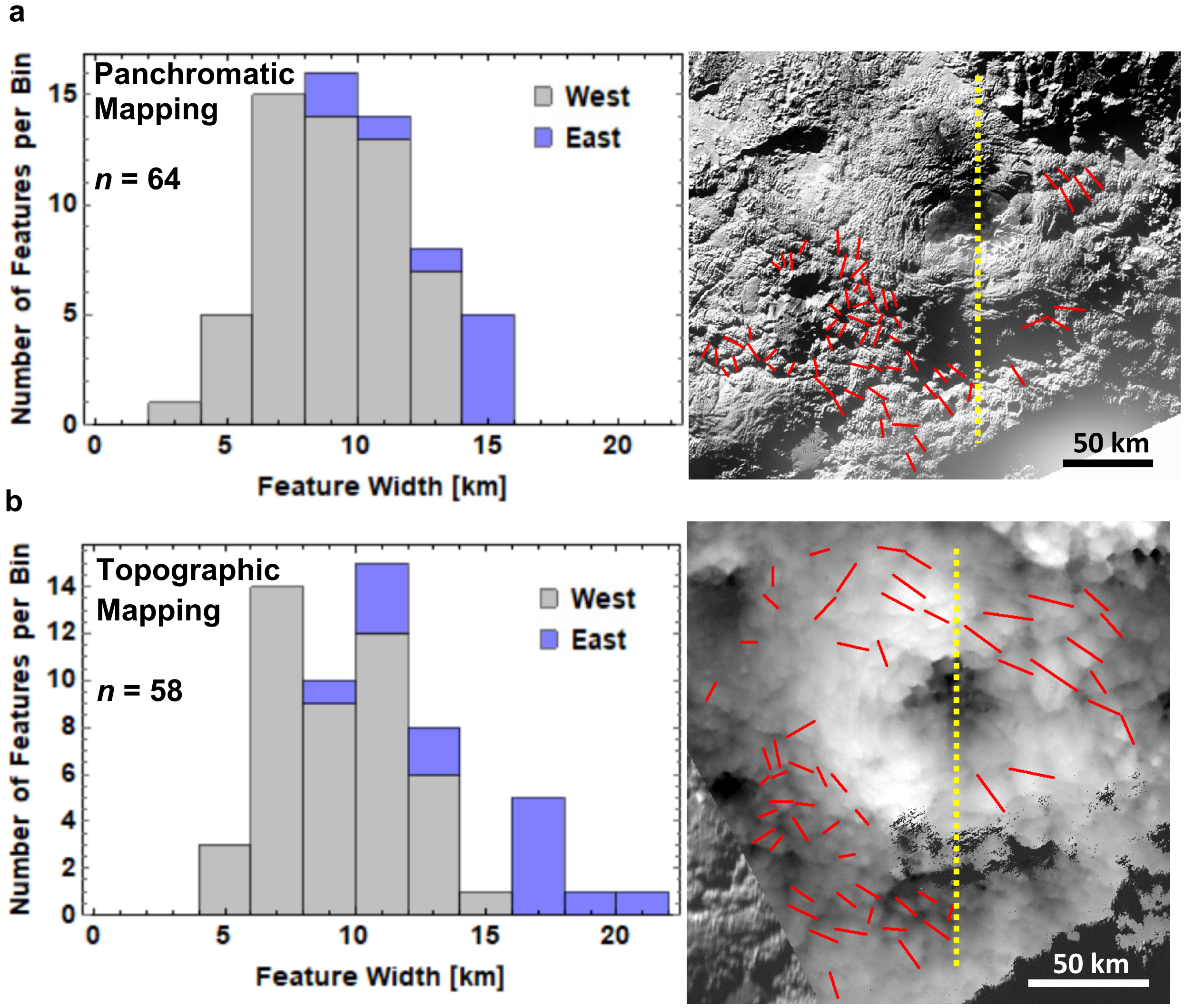

Supplementary Figure 5 | Hummocky terrain feature measurements. (a) Results for mapping performed on the panchromatic PEMV_P_MVIC_LORRI_CA dataset (315 m px^{-1}). **(b)** Results for mapping performed strictly with the topography. Vertical yellow, dashed line indicates the division between the eastern and western sides of the mapped region. Histogram bin widths were selected via the Sturges method. The longitude and latitude extents of both panels are similar to that of Supplementary Figure 4.

Supplementary Note 4: Additional close-up views of features in the Wright Mons region

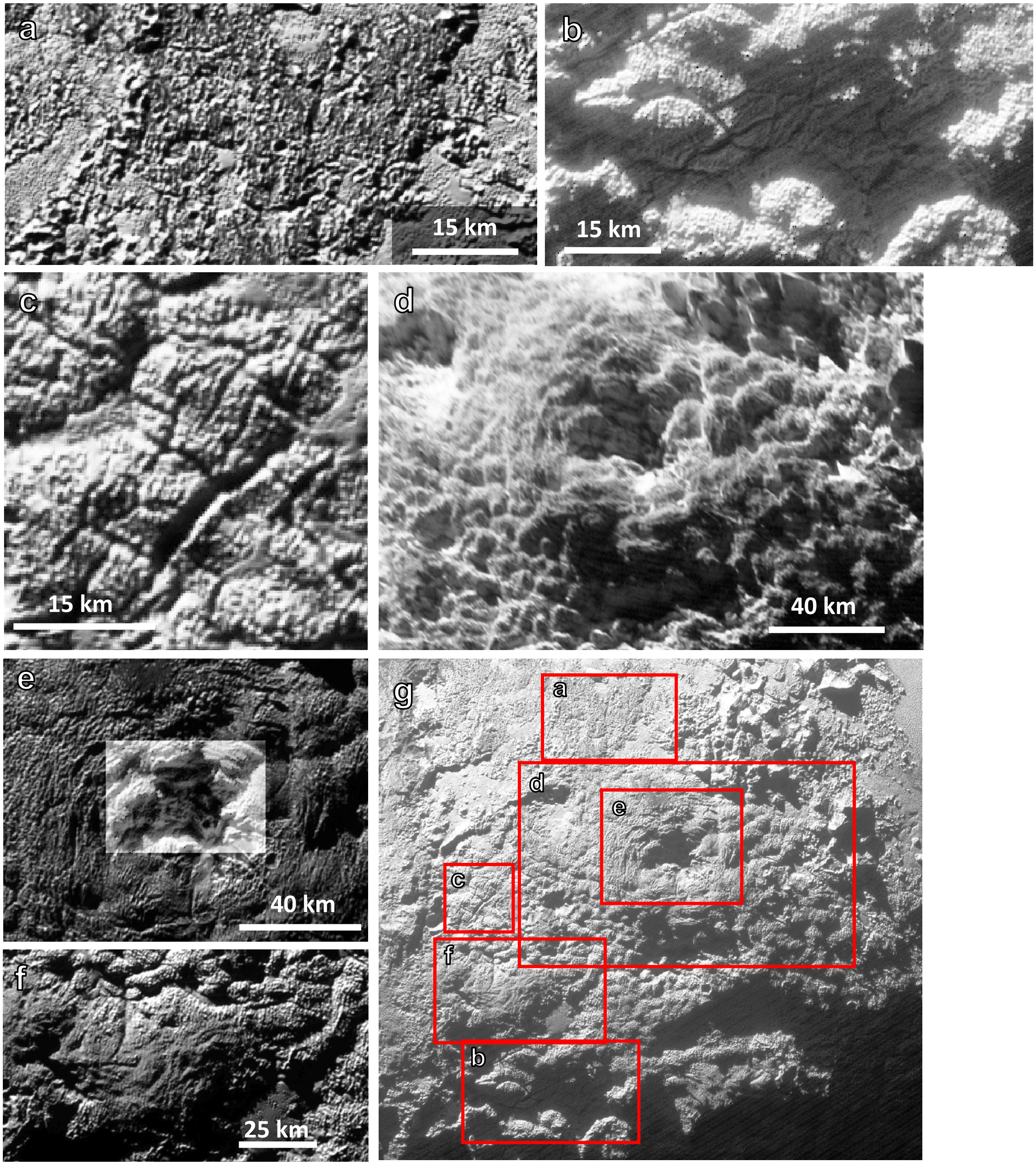

Supplementary Figure 6 | Additional views and image stretches of Wright Mons and the surrounding terrain. See text for details. Panels a-c and e-g are from the PEMV_P_MVIC_LORRI_CA observation (315 m/px; see Supplementary Table 1). Panel d is from the HiPhase_HiRes observation (340 m/px). Note that the striping in panel b is from the

instrument scanning, and is not a feature of the surface. Panel g shows the location of the features in this figure and is a subset of Supplementary Figure 1.

Supplementary Note 5: Comparison with Terrestrial and Martian Volcanic Profiles

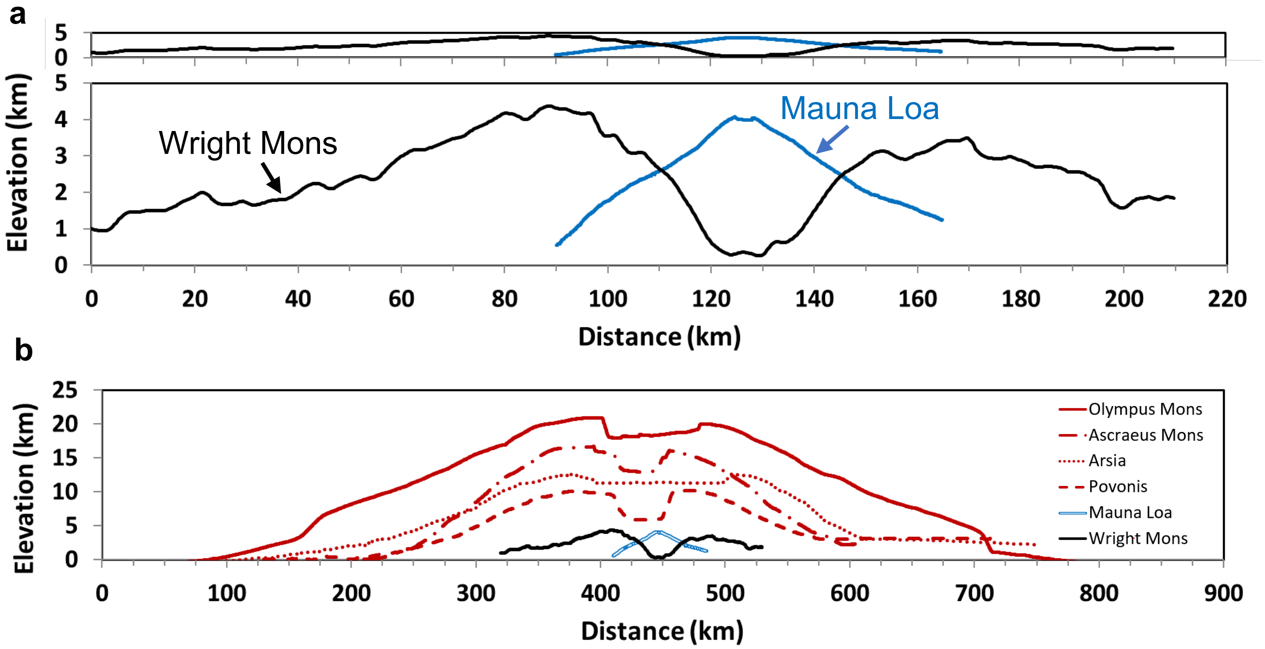

Supplementary Figure 7 | Topographic profile comparison. **(a)** Mauna Loa (subaerial portion only) compared with Wright Mons (~N-S through the feature), upper panel is without vertical exaggeration, and lower panel is shown at 10× vertical exaggeration. Mauna Lau continues for ~6 km below the ocean surface. This figure illustrates how Wright Mons is very dissimilar to Mauna Loa and that if Wright was originally more similar to a shield volcano it would have had to have lost >50% of its volume from the central region in order to attain its current appearance. (b) Martian shield volcanos from the Tharsis region provide additional examples of large volcanos, some with more advanced caldera collapse. Several of these also show signs of later embayment, thus they also may not represent the full original height of the features. The more typical collapse terraces can be seen in the Martian calderas. Note that none of these examples are scaled for gravity, they are shown at their original scales.

Supplementary Note 6: Comparison with Bladed Terrain on Pluto

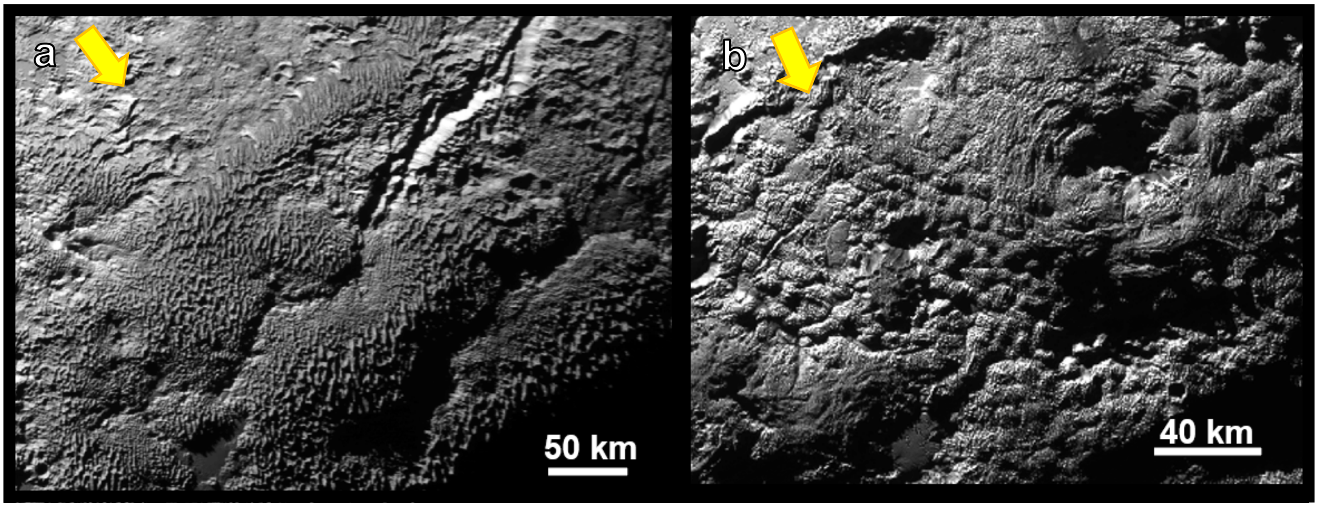

Supplementary Figure 8 | Comparison of Bladed Terrain and Wright Mons region. (a) The bladed terrain deposits form one of the highest elevation areas on Pluto (on the far eastern side of the hemisphere observed by New Horizons during closest approach) and are hypothesized to be a sprawling, concentrated deposit of methane ice, with the bladed texture forming due to sublimation of methane [4]. It may also exist in large areas on the "far side" of Pluto that was only observed at low resolution by New Horizons [5]. (b) Although Wright Mons has several different scales and styles of textures, it is not covered by the distinctive blades that characterize the bladed terrain. The longitude and latitude extents are as follows: panels a ~220–243°E and ~10–26°N; panel b ~165–175°E and ~19–26°S. The large arrows in the upper left indicate the approximate direction of the incoming light.

Supplementary Note 7: Comparison with Funiscular Terrain on Enceladus and Pahoehoe on Earth

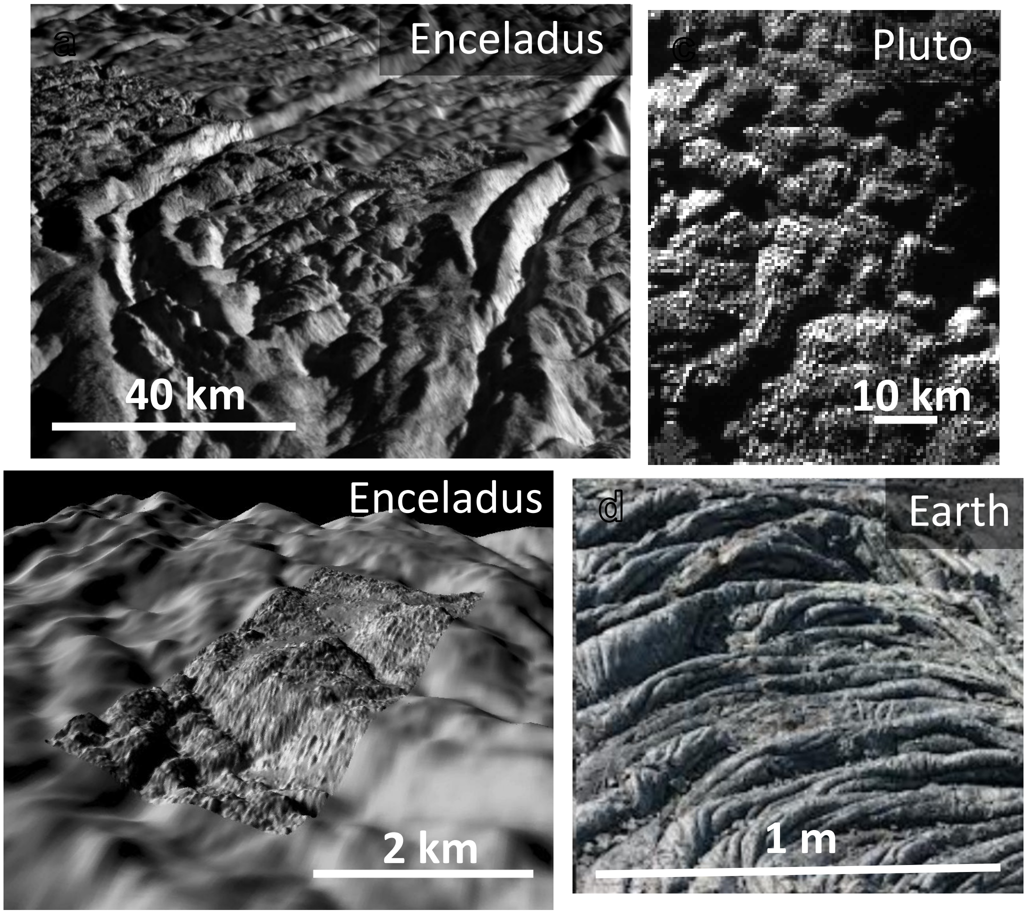

Supplementary Figure 9 | Funiscular terrain and pahoehoe compared to Pluto. (a-b) Perspective views of funiscular terrain on Enceladus showing the lumpy material between the tiger stripes has a somewhat consistent size/wavelength (images courtesy NASA/JPL/Space Science Institute/Universities Space Research Association/Lunar & Planetary Institute and can be found at https://photojournal.jpl.nasa.gov/catalog/PIA12208 and https://photojournal.jpl.nasa.gov/catalog/PIA10350. (c) Hummocky/undulatory terrain on Pluto. (d) Pahoehoe lava texture on Earth.

Supplementary Note 8: Volume Estimate

The volume of the main topographic rise of the feature outlined as Wright Mons (as shown in Supplementary Figure 10) was measured from the highest elevations on the feature down to an elevation of -1 km in the DEM [1]. An elevation of -1 km is near where the northern base of the large rise transitions to the flatter surrounding terrain, but there is not a clear topographic boundary

to the feature on the southern side. This yielded an estimate of ~2.4 x 10^4 km^3 for just this area. Using a lower bound elevation of -0.5 km or -1.5 km (0.5 km higher or lower than our original choice of -1 km) yields volume estimates of ~1.6 x 10^4 km^3 or ~3.3 x 10^4 km^3, respectively (for the same feature outline as shown in Supplementary Figure 10). The uncertainty in the feature outline and the uncertainty in the location of the base of the feature (i.e., how one arbitrarily defines the extent and base location of Wright Mons) is larger than the vertical precision in the DEM (90 m) utilized for this measurement.

Here we provide the volume of this feature as an example. This volume is similar in magnitude to that of the Hawaiian volcano Mauna Loa, estimated to have a volume of ~6-8 x 10^4 km^3 for the total of the subaerial and submerged portions [6]. The resurfacing of the area in general and the creation of the other large rises (e.g., the medial montes region and Piccard Mons) would require a considerable additional volume of material.

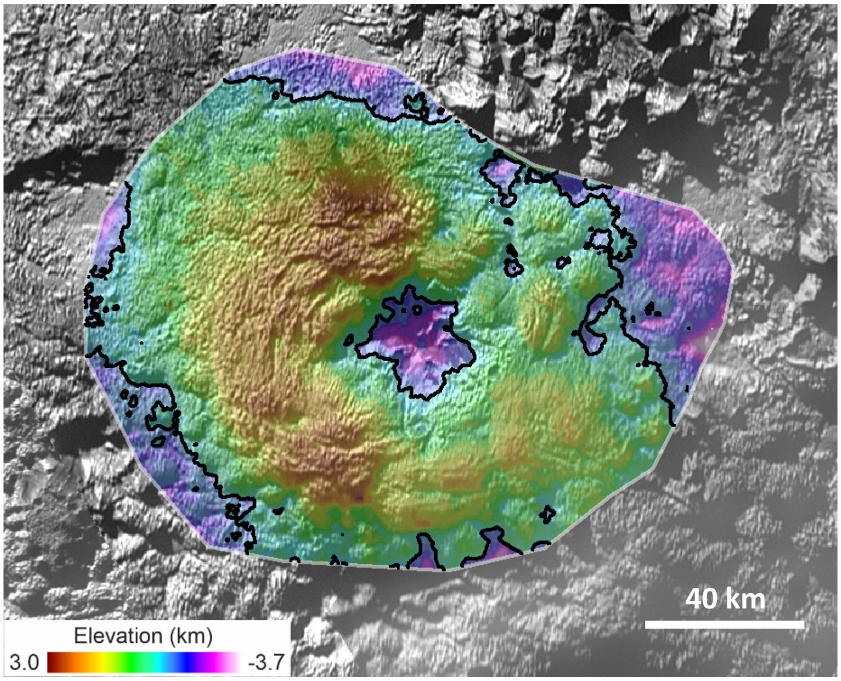

Supplementary Figure 10 | Wright Mons volume estimate. The white outline shown here is an approximate boundary for the feature that has been named Wright Mons. The southern part of the feature does not have a clear boundary with the adjacent large rises, but for the sake of measurement we have outlined one region here. The black elevation contour is placed at -1 km in the DEM and was used for the approximate lower boundary of the feature in order to estimate the volume of material needed to create Wright Mons (see text). The longitude and latitude extents of this image are ~167–176°E and ~17–26°S.

Supplement References

1. Schenk PM, Beyer RA, McKinnon WB, Moore JM, Spencer JR, White OL*, et al.* Basins, fractures and volcanoes: Global cartography and topography of Pluto from New Horizons. *Icarus* 2018, **314:** 400-433.
2. Reuter DC, Stern SA, Scherrer J, Jennings DE, Baer JW, Hanley J*, et al.* Ralph: A Visible/Infrared Imager for the New Horizons Pluto/Kuiper Belt Mission. *Space Science Reviews* 2008, **140:** 129-154.
3. Cheng AF, Weaver HA, Conard SJ, Morgan MF, Barnouin-Jha O, Boldt JD*, et al.* Long-Range Reconnaissance Imager on New Horizons. *Space Science Reviews* 2008, **140:** 189-215.
4. Moore JM, Howard AD, Umurhan OM, White OL, Schenk PM, Beyer RA*, et al.* Bladed Terrain on Pluto: Possible origins and evolution. *Icarus* 2018, **300:** 129-144.
5. Stern SA, White OL, McGovern PJ, Keane JT, Conrad JW, Bierson CJ*, et al.* Pluto's Far Side. *Icarus* 2021, **356:** 113805.
6. Kaye GD. Using GIS to estimate the total volume of Mauna Loa Volcano, Hawai`i. The Geological Society of America - Cordilleran Section, 98th Annual Meeting; 2002; Corvallis, Oregon; 2002.
